# Near-Field Radiative Heat Transfer Modulation with an Ultrahigh Dynamic Range through Mode Mismatching

**DOI:** 10.1021/acs.nanolett.2c01286

**Published:** 2022-09-26

**Authors:** Kezhang Shi, Zhaoyang Chen, Yuxin Xing, Jianxin Yang, Xinan Xu, Julian S. Evans, Sailing He

**Affiliations:** † Centre for Optical and Electromagnetic Research, National Engineering Research Center for Optical Instruments, Zhejiang University, Hangzhou 310058, China; ‡ Centre for Optical and Electromagnetic Research, ZJU-SCNU Joint Center of Photonics, South China Academy of Advanced Optoelectronics, South China Normal University, Guangzhou 510006, China; § Shanghai Institute for Advanced Study, Zhejiang University, Shanghai 201203, China; ∥ Department of Electromagnetic Engineering, School of Electrical Engineering, Royal Institute of Technology, Stockholm S-100 44, Sweden

**Keywords:** modulation of near-field radiative heat transfer, graphene, surface plasmon polaritons coupling, mode mismatching, fluctuational electrodynamics, radiative heat transfer measurement

## Abstract

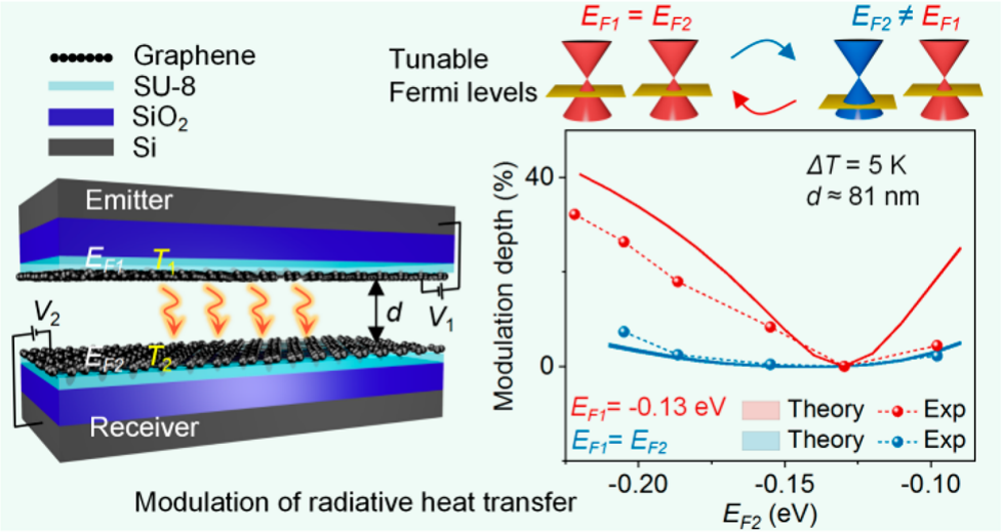

Modulating near-field radiative heat transfer (NFRHT) with a high dynamic range is challenging in nanoscale thermal science and engineering. Modulation depths [(maximum value – minimum value)/(maximum value + minimum value) × 100%] of ≈2% to ≈15.7% have been reported with matched modes, but breaking the constraint of mode matching theoretically allows for higher modulation depth. We demonstrate a modulation depth of ≈32.2% by a pair of graphene-covered SU8 heterostructures at a gap distance of ≈80 nm. Dissimilar Fermi levels tuned by bias voltages enable mismatched surface plasmon polaritons which improves the modulation. The modulation depth when switching from a matched mode to a mismatched mode is ≈4.4-fold compared to that when switching between matched modes. This work shows the importance of symmetry in polariton-mediated NFRHT and represents the largest modulation depth to date in a two-body system with fixed gap distance and temperature.

Thermal radiation, with spectral and power density properties described by Planck’s Law and the Stefan–Boltzmann Law, is one of the known noncontact heat transfer modes in vacuum.^1,2^ Classical physics predicts that a perfect thermal emitter operates at the blackbody (BB) limit. Fluctuational electrodynamics has demonstrated that evanescent modes, including plasmon and phonon polaritons, allow large near-field radiative heat transfer (NFRHT) far beyond the limit.^3−14^ Compared to the general far-field broadband radiative spectrum, NFRHT is primarily dominated by the resonance coupling modes between two close objects, allowing for active control of the spectrum^11,15^ and energy transfer.^6,16^

Dynamic modulation of NFRHT requires changing the optical responses of the emitter and receiver to modulate the radiative heat flux. Previous works^17−20^ have shown that a phase-change material (VO_2_) allows for thermal radiation modulation due to its insulator-to-metal transition. However, the modulation requires temperature variation. Graphene has highly tunable surface plasmon polaritons (SPPs) related to its Fermi level in a linear Dirac band, which makes it an ideal thermal modulator with external stimuli at a fixed operating temperature.^21−23^ Altering the free carrier states of a van der Waals’ heterostructure also allows for exotic nanoscale phenomena such as tunable Mott insulator,^24^ nanoimaging in an infrared-waveguide,^25^ and efficient Fizeau drag from Dirac electrons,^26^ etc. Recent experimental works have demonstrated that graphene plasmons enable giant radiative heat transfer at the nanoscale.^5,6,12^ Graphene with different Fermi levels supports tunable SPPs and accounts for different radiative heat flux. A high dynamic range modulation of NFRHT allows for a high signal-to-noise ratio (SNR) enabling potential applications in thermal switches and communication. Thomas et al. have shown an electronic modulation depth of ≈2% [defined by (maximum value–minimum value)/(maximum value + minimum value) × 100%] with a pair of graphene sheets on Al_2_O_3_/SiO_2_ substrate.^21^ The Fermi levels of the two graphene sheets are assumed to be equal since the samples are in conductive contact. Our recent work has shown that the modulation depth could reach ≈15.7% with stacked graphene layers at similar Fermi levels.^6^ However, the modulation effect is limited by the matched resonance modes between the identical graphene sheets, as the graphene on the emitter and receiver have similar Fermi levels.

Here, we present a significant modulation improvement by a pair of graphene/SU8 heterostructures at a gap distance of ≈80 nm. Back-gated tuning was employed for the graphene Fermi level control. The mismatched SPPs due to different Fermi levels of the two graphene sheets allow for a much smaller heat flux compared to the matched SPPs case. The measured maximum modulation depth could reach ≈32.2% and is ≈4.4-fold compared to that of the matched case. This experimental work represents the largest modulation depth ever reported for the radiative heat transfer in a two-body planar system with a fixed gap distance and operating temperature. The mode-mismatch-induced high-efficiency NFRHT modulation should inspire potential applications of thermal switches,^22,27,28^ thermal communication, and is suitable for tunable plasmon- or phonon-induced thermal or photonic regulation.

We study the NFRHT between a pair of graphene-covered SU8 heterostructures on SiO_2_/Si substrates (marked as Gr/SU8, Figure 1). The thickness of the SU8 is chosen to be 90 nm to reduce the influence of phonon polaritons from the substrate. Based on the fluctuational electrodynamics, the net radiative heat flux between the emitter and receiver (with temperatures *T*_1_ and *T*_2_, respectively) at a gap distance *d* is calculated by^29−33^
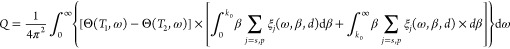
1where Θ(*T*,ω) = *ℏω*/[exp(*ℏω*/*k*_B_*T*) – 1] is the mean energy of the Planck thermal harmonic oscillators without zero point energy. *ℏ* is the reduced Planck constant and *k*_B_ is the Boltzmann constant. ξ_*j*_ represents the energy transmission coefficient between the emitter and receiver (considering both *s*- and *p*-polarization modes):
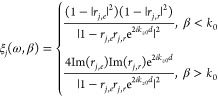
2where *k*_z0_ is the *z*-component of the wave vector in vacuum (*k*_0_). *r*_*j,e*_ and *r*_*j,r*_ are the Fresnel reflection coefficients of the emitter and receiver, respectively. ξ_*p*_ with β > *k*_0_ represents the photon tunneling probability of the *p*-polarized evanescent modes.

**Figure 1:**
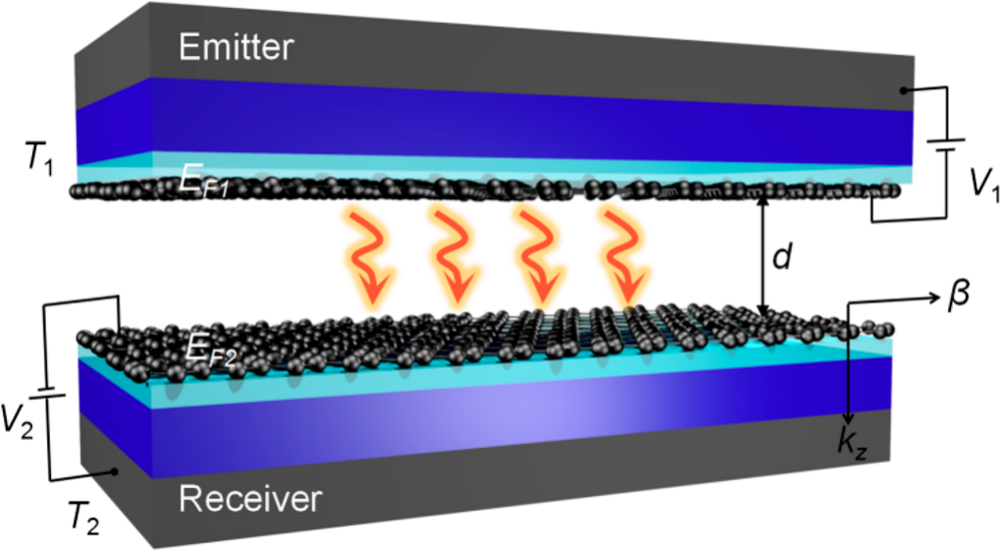
Schematic illustration of a pair of graphene-covered SU8 heterostructures (on SiO_2_/Si substrates) separated by a vacuum gap distance *d*. *T*_1_ and *T*_2_ are set to 308.15 and 303.15 K, respectively. A back-gated tuning method with applied bias voltages *V*_1_ and *V*_2_ is employed to control the Fermi levels of the two graphene sheets. The red arrows illustrate the net radiative heat flux flowing from the emitter to the receiver.

For a homogeneous medium with finite thickness, the reflection coefficients become^29,32^
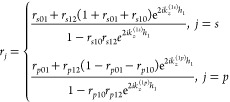
3where *h*_1_ is the thickness of layer one and *r*_*s*(*p*),01_ is the Fresnel reflection coefficient at the interface between layer 0 and layer 1 for *s*- or *p*-polarization modes:
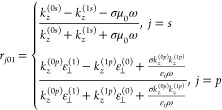
4where , , *n* = 0, 1, 2 is the number of the layers, ε_⊥_^(*n*)^ and ε_||_^(*n*)^ are the perpendicular and parallel components of the relative dielectric tensor. Here, ε_⊥_^(*n*)^ = ε_||_^(*n*)^ is set for the isotropic medium. Graphene was treated as surface current with complex conductivity σ:^34,35^
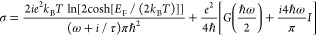
5where
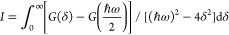
6
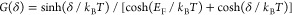
7The left and right terms in eq 5 account for the intraband and interband electron transitions, respectively. *E*_F_ is the Fermi level of graphene. τ = 100 fs related to the carrier–carrier intraband collisions and phonon emission is used in our calculations for the collision time.^5,6,12,36,37^ The dielectric function of the vacuum-like SU8 material was modeled as multiple Lorentz–Drude oscillators,^6,38^ while the dielectric function of SiO_2_ was taken from ref (39). The Si substrate was omitted due to its negligible contribution to the NFRHT.

The photon tunneling probability ξ_p_ of the evanescent modes between the emitter and receiver at a gap distance of 80 nm is calculated and shown in Figure 2a,b. Strong coupling modes arising from the matched SPPs could be observed when the graphene Fermi levels of the receiver (*E*_F2_) and emitter (*E*_F1_) are both −0.13 eV (Figure 2a), corresponding to our experiment with bias voltages (*V*_2_, *V*_1_) = (35, 35) V. The SPP coupling modes are slightly deviated from the ideal dispersion curves of a pair of suspended graphene sheets (dashed-dotted lines) due to the impact of the SU8 spacers and the SiO_2_ substrates. Typical phonon polaritons from the SiO_2_ substrate support the near-unity ξ_p_ around wavelengths of 8.56 and 19.9 μm, but have no contribution to the heat flux modulation. The yellow-dashed lines correspond to the occupation factor Θ(*T*_1_,ω) – Θ(*T*_2_,ω) in arbitrary units. The near-unity ξ_p_ overlaps the dispersion curves of the SPP coupling modes of two Gr/SU8 heterostructures (not shown). The coupled modes split into two branches at a lower β but merge at a larger β, as the larger loss (at large β) in the vertical direction enables rapid attenuation of the SPP modes and prevents the interaction between the emitter and receiver. These matched SPP coupling modes are supported at the mid- and far-infrared regions with a larger occupation factor and are the dominant contributor to the radiative heat flux. Compared with changing the graphene Fermi levels synchronously (e.g., change both *E*_F2_ and *E*_F1_ to −0.22 eV), producing dissimilar Fermi levels is a more effective way to pursue an improved modulation depth. When only the graphene Fermi level of the receiver changes to −0.22 eV (Figure 2b), the SPPs arising from the emitter and receiver contribute less to the near-unity ξ_p_. Due to the mismatched SPP modes, the resonance modes are decoupled around 1.0 × 10^14^ and 1.5 × 10^14^ rad/s with large β. In contrast, the phonon polaritons modes remain unchanged due to the identical SiO_2_ substrates on both sides. The spectral heat flux (after integration over β) between the Gr/SU8 heterostructures shows the influence of the graphene SPPs with identical or dissimilar Fermi levels (Figure 2c). When *E*_F2_ = *E*_F1_ = −0.13 eV (case I), the spectrum attributed to the matched strong SPPs covers a broad frequency region with the highest value. A broader spectrum with lower spectral heat flux (compared to case I) could be observed when *E*_F2_ = *E*_F1_ = −0.22 eV (case II). However, the spectral heat flux decreases dramatically when *E*_F2_ is not equal to *E*_F1_, where *E*_F2_ = −0.22 and *E*_F1_ = −0.13 eV (case III). The spectrum in case III shares a similar frequency region with case I, but it has smaller intensity due to the mismatched resonance modes. The radiative heat flux reaches 1.34 × 10^4^, 1.19 × 10^4^, and 0.57× 10^4^ W/m^2^ for cases I, II, and III, respectively. This leads to a remarkable improvement of the NFRHT modulation depth from case I to case III, where the modulation depth is 40.3% [calculated by (radiative heat flux of case I – radiative heat flux of case III)/(radiative heat flux of case I + radiative heat flux of case III) × 100%] and is ≈6.7-fold compared to that from case I to case II. Figure 2d gives the contour map of the radiative heat flux normalized with corresponding BB limit with variable *E*_F1_ and *E*_F2_. For different *E*_F1_ at a fixed *E*_F2_, the radiative heat flux arrives at a maximal value with two similar *E*_F_. This confirms the importance of the symmetry of the polariton-mediated NFRHT system. White-dashed line shows the cases with matched coupling SPP modes. Case I with η = 416 is quite close to the peak point at *E*_F2_ = *E*_F1_ = −0.14 eV. The white-dashed-dotted line highlights the cases corresponding to the mismatched resonance modes. Finding a point with smaller heat flux based on the mismatched graphene Fermi levels allows for a higher modulation depth. In this work, the radiative heat flux from case I to case II, and case I to case III were measured.

**Figure 2:**
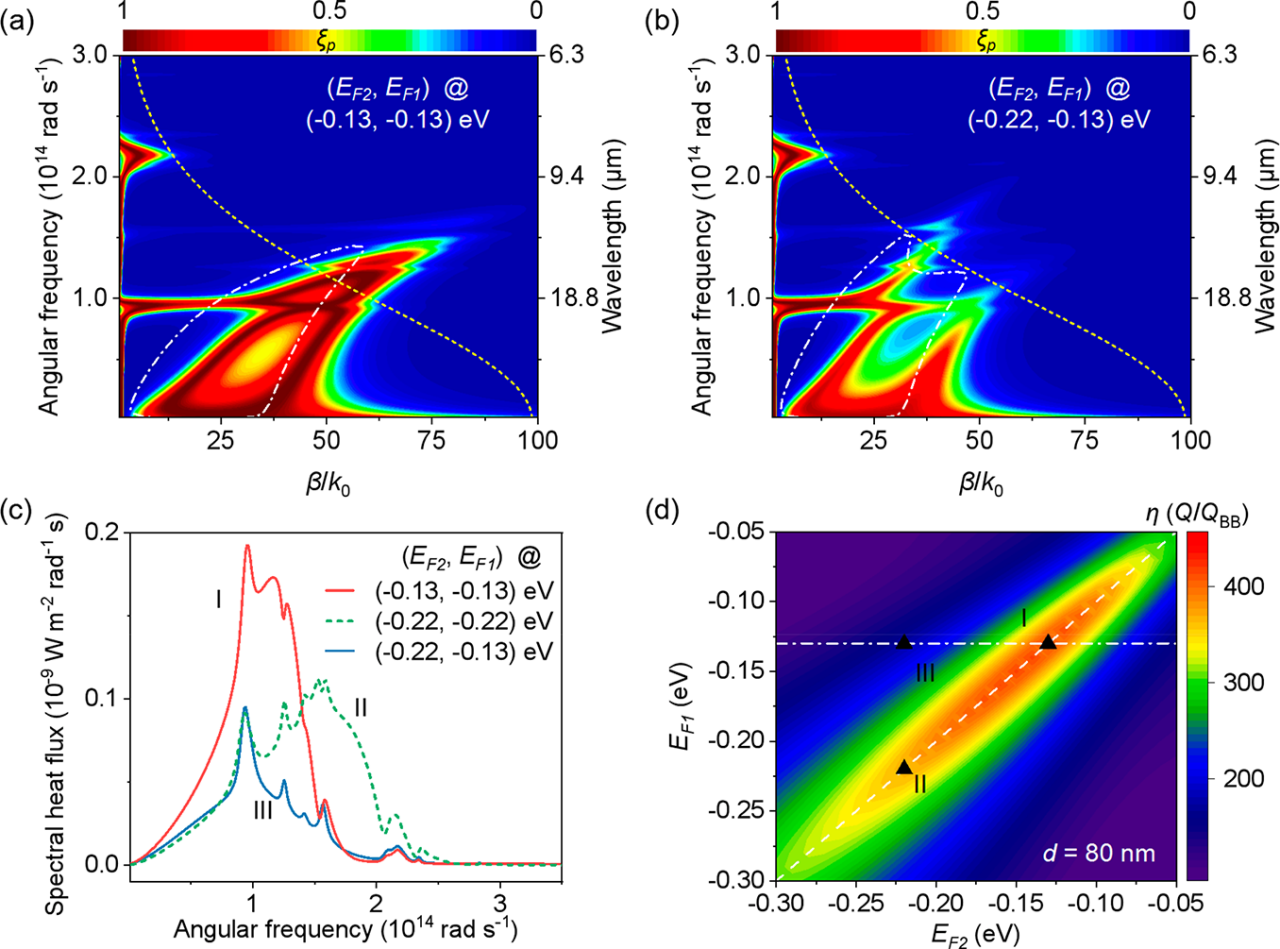
Calculation and analysis of the NFRHT modulation of Gr/SU8 heterostructures at *d* = 80 nm with *T*_1_ = 308.15 K and *T*_2_ = 303.15 K. Contour maps of ξ_p_ for a pair of Gr/SU8 heterostructures (on SiO_2_/Si substrates) with *E*_F2_ = *E*_F1_ = −0.13 eV for (a) and *E*_F2_ = −0.22 eV, *E*_F1_ = −0.13 eV for (b). The yellow-dashed lines in (a) and (b) correspond to the occupation factor Θ(*T*_1_,ω) – Θ(*T*_2_,ω) in arbitrary units, while dashed-dotted lines are the dispersion curves of the coupled SPPs of the two suspended graphene sheets. (c) Spectral heat flux of Gr/SU8 heterostructures for three cases of graphene Fermi levels (*E*_F2*,*_*E*_F1_), where *E*_F2_ = *E*_F1_ = −0.13 eV for case I (red-solid line), *E*_F2_ = *E*_F1_ = −0.22 eV for case II (green-dotted line), and *E*_F2_ = −0.22 eV, *E*_F1_ = −0.13 eV for the mismatched case III (blue-solid line). (d) Contour map of the radiative heat flux *Q* normalized with the corresponding BB limit with *E*_F2_ and *E*_F1_ ranging from −0.3 eV to −0.05 eV. The white-dashed-dotted line corresponds to the cases of mismatched resonance modes, while the white-dashed line (along the diagonal direction) represents the cases of the matched SPPs.

The emitter and receiver consist of single-layer graphene-covered SU8 heterostructures on the 300 nm-thick SiO_2_ on Si substrates (see Supporting Information (SI) section 2). Eight identical SU8 nanopillars with thickness of ≈90 nm were fabricated on the surface of the graphene sheets for the receiver (Figure 3a). A back-gated device was employed to tune the Fermi levels of graphene. Figure 3b shows the side view of the receiver within the active area (white-dashed square with *L* = 3 mm in Figure 3a). A positive (negative) *V*_g_ – *V*_n_ induces electron (hole) doping,^40,41^ allowing an excess-electron surface concentration of *n* = η_c_(*V*_g_ – *V*_n_), where *V*_g_ is the gate-tuning voltage, *V*_n_ is the voltage at the charge neutral point, and η_c_ is a coefficient related to the back-gated structure with gate insulators composed of 90 nm thick SU8 and 300 nm thick SiO_2_ (see SI section 1). The graphene Fermi levels could be obtained by *E*_F_ = sgn(*n*) *ℏV*_F_(π |*n*|)^1/2^/e (unit: eV), where sgn(*x*) is the sign of *x* and *V*_F_ = 1 × 10^6^ m/s is the Fermi velocity of graphene.^40−44^ The emitter was pressed on the receiver in a cross shape and separated by the SU8 nanopillars (Figure 3c). A total of 95 g mass above the emitter and two fixed posts were used to strengthen the contact and mechanical stability of the system (Figure 3d).^6,10,12,33^ The gap distance was estimated within a range from ≈79 to ≈83 nm (i.e., at an average value of ≈81 nm) based on the one-dimensional linear elastic analysis^6^ (SI section 4). Figure 3e illustrates the equivalent thermal circuit of the system. *P*_sum_ detected by the heat flux sensor (HFS) is the sum heat power of *P*_c_ and *P*_r_, where *P*_r_ is the near-field radiative heat power including the contribution of both propagating waves and evanescent waves. The measured radiative heat flux could be obtained by *Q* = (*P*_sum_ – *P*_c_)/*A*, where *A* is the active area of 3 × 3 mm^2^. *P*_c_ is the heat conduction from the eight SU8 nanopillars and is calculated based on Fourier’s Law (SI section 5).

**Figure 3:**
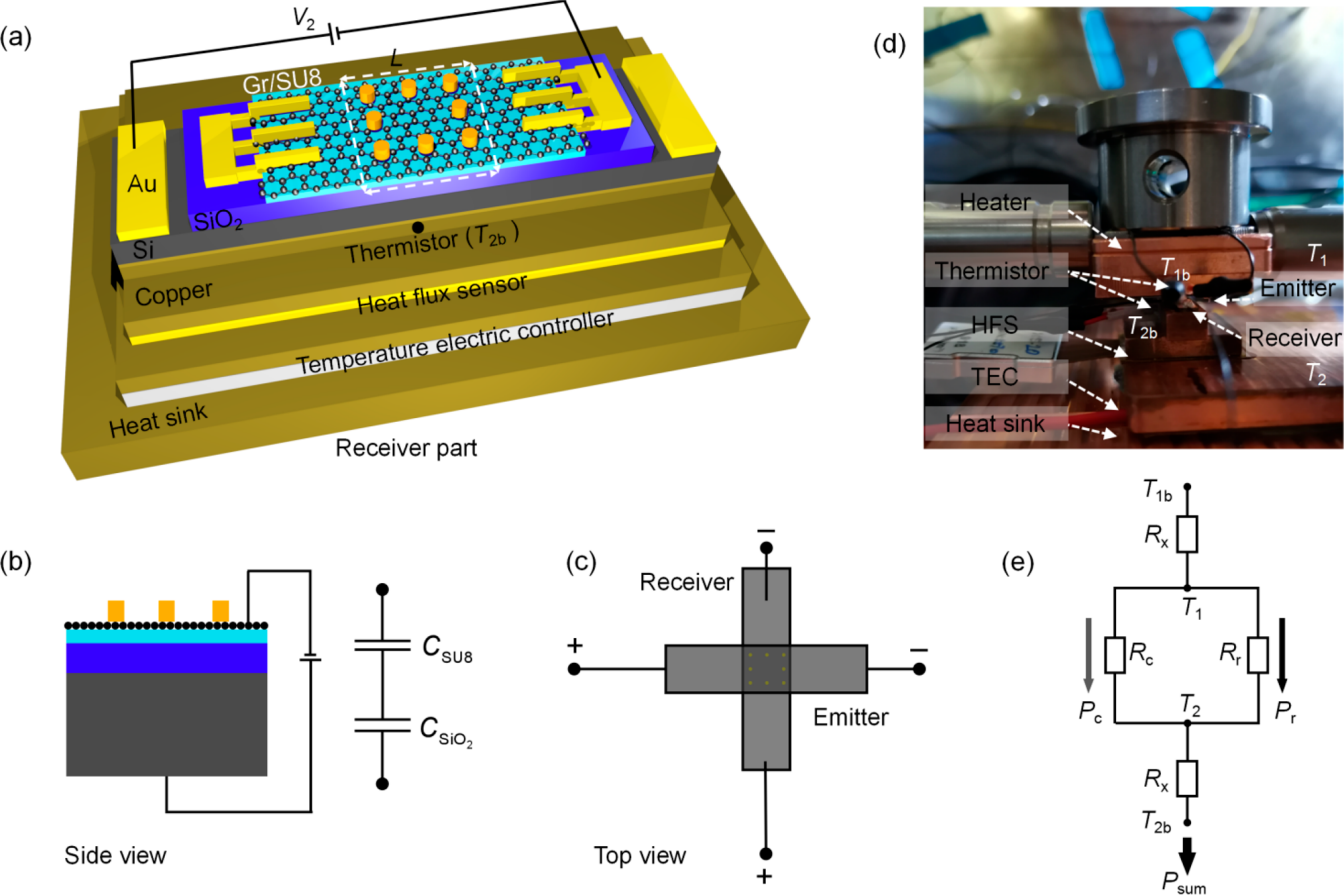
Schematic illustrations of the NFRHT experimental setup. (a) Schematic diagram of the receiver part. White-dashed square shows the active area of 3 × 3 mm^2^. Au/Ti electrodes for both emitter and receiver are conductively contacted with the graphene sheets to control the Fermi level by the external electrostatic field. (b) Side view of the receiver. The receiver (or emitter) was modeled as two capacitors in series, where *C*_su8_ and *C*_SiO_2__ are the capacitances per unit area of the SU8 spacer and SiO_2_ dielectric, respectively. The graphene Fermi level was determined by the equivalent capacitance model. (c) Top view of the emitter and receiver. The conductive wires with positive and negative signs correspond to the two independent back-gated devices. (d) Photo of the NFRHT experimental device in a vacuum chamber. The heater and temperature electric controller (TEC) were used to control the temperature of the emitter and receiver, respectively. The embedded thermistors were used to measure the backside temperature *T*_2b_ (*T*_1b_) of the receiver (emitter). See more discussion in SI section 3. (e) Equivalent thermal circuit of the NFRHT experimental device. *P*_sum_ is the sum of the heat powers contributed by the heat conduction portion (*P*_c_) and the radiative portion (*P*_r_). *R*_c_ and *R*_r_ are the thermal conduction resistance and equivalent thermal radiation resistance, respectively. *T*_1_ and *T*_2_ are the top surface temperatures of the emitter and receiver, respectively, estimated by the measured sum *R*_*x*_ (SI section 3) of the thermal resistances of the sample, thermal conductive adhesive, and the copper carrier. *T*_2_ was maintained at 303.15 K, while *T*_1_ was 308.15 K unless otherwise specified.

The radiative heat flux shown in Figure 4a was measured at *T*_2_ = 303.15 K with temperature difference *ΔT* of 5 K at a gap distance of ≈81 nm. When no bias voltages are applied to the emitter and receiver, i.e., (*V*_2_, *V*_1_) = (0, 0) V, *E*_F2_ and *E*_F1_ are calculated to be −0.205 eV according to the measured radiative heat flux. According to the back-gated method, other bias voltages of (10, 10), (25, 25), (35, 35), and (45, 45) V correspond to the graphene Fermi levels of −0.187, −0.155, −0.13, and −0.098 eV respectively. The Fermi levels become closer to the Dirac point with higher positive voltages, indicating the hole doping of the graphene sheets. When *E*_F2_ = *E*_F1_ = −0.13 eV, the matched SPPs allow for the broadband near-unity ξ_p_ within the desired mid- and far-infrared region, producing the best performance among all measured cases. The radiative heat flux reaches 1.43 × 10^4^ W/m^2^ and is ≈441-fold of the corresponding BB limit. When *E*_F2_ = *E*_F1_ = −0.205 eV, the enhancement (≈ 381-fold with respect to the BB limit) is relatively robust, as the SPP modes of the emitter and receiver are still matched, despite the blue-shift to a higher frequency region with less occupation factor (similar to the spectral heat flux of case II in Figure 2c). For the mismatched cases, the bias voltage of the emitter *V*_1_ is fixed at 35 V while the bias voltage of the receiver *V*_2_ is set to −10, 0, 10, 25, 35, and 45 V. Remarkable modulation of the radiative heat flux was observed when the bias voltage of the receiver became different from that of the emitter. Here the minimum radiative heat flux (≈ 220-fold with respect to the BB limit) was observed at (*V*_2_, *V*_1_) = (−10, 35) V, where the graphene Fermi levels are dissimilar [i.e., (*E*_F2_, *E*_F1_) = (−0.22, −0.13) eV]. When *V*_2_ increases to 35 V again, the radiative heat flux can still reach a peak value of 1.39 × 10^4^ W/m^2^ with enhancement of ≈429-fold of the BB limit, indicating the robustness of the tuning devices. Figure 4b illustrates the modulation depths [(measured maximum value–other measured value)/(measured maximum value + other measured value) × 100%] of the matched and mismatched cases, respectively. The maximum modulation depth achievable with only matched cases is ≈7.3% going from (*E*_F2_, *E*_F1_) = (−0.13, −0.13) eV to (*E*_F2_, *E*_F1_) = (−0.205, −0.205) eV, but the modulation depth is ≈26.3% when only changing *E*_F2_ to −0.205 eV. The maximum modulation depth from the matched case to a mismatched case reaches ≈32.2% when tuning *E*_F2_ to −0.22 eV by applying −10 V bias voltage to the receiver. It could potentially be further improved when other larger negative voltages are applied. Considering the breakdown of the capacitor-like samples, only −10 V was investigated in this work. Construction of the mismatched resonance modes plays a significant role in the NFRHT modulation improvement. Switching the symmetry of the polariton-mediated near-field system gives promising high-efficiency modulation in thermal radiation. The modulation depth of ≈32.2% is ≈4.4-fold compared to that for the matched cases and is 16.1-fold of the previous report on graphene-based heterostructures.^21^ The modulation of the radiative heat flux at another *ΔT* of 3 K was also investigated in Figure 4c. The time-varying heat flux were recorded by the HFS when changing the bias voltages from (*V*_2_, *V*_1_) = (35, 35) V to (25, 35) V repeatedly. The average modulation depth of ≈7% is similar to that with *ΔT* = 5 K in Figure 4b (≈ 8.3%). Small *ΔT* (like that in this work) is more likely required in potential applications such as thermal communication, since the state of the thermal equilibrium will be easier to achieve after switching the graphene Fermi levels, hence a faster response time. The results show good repeatability and robustness of our devices for the NFRHT modulation. The sample in this work consists of a single-layer graphene-covered SU8 heterostructure, which is simpler and easier to fabricate, and allows for larger modulation depth (with mismatched modes) than that of the previous work^6^ with multilayer structure (only matched modes are considered). The physical mechanism in ref (6). is that multilayer systems allow many branches in *k*-space to provide stronger NFRHT and modulation with the tuning of only one Fermi level was used for the optimal radiative heat flux. This work focused on the ultrahigh dynamic modulation and the essential physics is that independent tuning of two different Fermi levels allows a massive improvement in modulation depth. In addition, the determination of the graphene Fermi level is significantly improved by the equivalent capacitance model related to the back-gated tuning devices.

**Figure 4:**
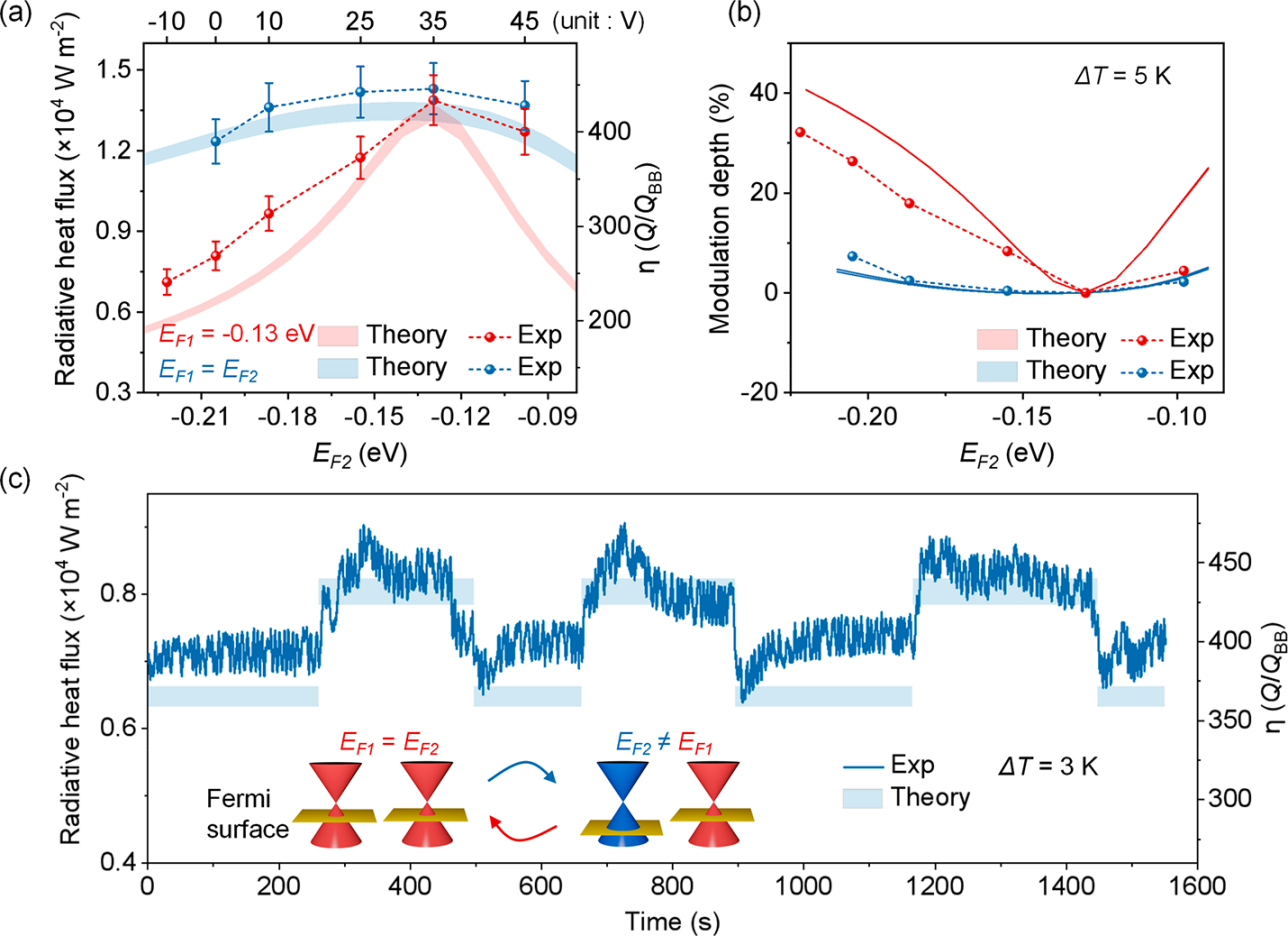
Measurements and analysis of the NFRHT modulation of the Gr/SU8 heterostructures at the gap distance of ≈81 nm with different bias voltages. The colored bands in all panels are the corresponding theoretical calculation with *d* from 79 to 81 nm. (a) Radiative heat flux varies with the bias voltages (graphene Fermi levels) at temperature difference *ΔT* = 5 K for the matched cases (blue lines) and the mismatched cases (red lines). Each point of the curves in (a) and (b) is an average value from three possible active areas (2.9 × 2.9 mm^2^, 3 × 3 mm^2^, and 3.1 × 3.1 mm^2^) after measuring for four times from the external heat flux meter. Error bars were plotted due to the uncertainty of the active area (see SI section 6). (b) Modulation depth of different graphene Fermi levels corresponding to the matched and mismatched cases in (a). The modulation depth is calculated by (measured maximum value–other measured value)/(measured maximum value + other measured value) × 100%. (c) Time-varying heat flux of the Gr/SU8 heterostructures at *ΔT* = 3 K. Blue-solid line is the measured radiative heat flux and the blue bands are the theoretical prediction. Inset shows the illustrations of the tunable graphene Fermi levels of the emitter and receiver due to different applied bias voltages.

The modulation depth versus variable vacuum gap distance *d* within a range from 50 to 500 nm is theoretically investigated (Figure 5). The black-dashed line shows the gap-dependent radiative heat flux between two identical Gr/SU8 heterostructures with (*E*_F2_, *E*_F1_) = (−0.13, −0.13) eV, and the dashed-dotted line indicates the mismatched cases with (*E*_F2_, *E*_F1_) = (−0.22, −0.13) eV. The modulation depths (red-solid line) are calculated by the radiative heat flux when switching the graphene Fermi levels from (−0.13, −0.13) eV to (−0.22, −0.13) eV at the corresponding *d*. The modulation depth can be improved to 43.6% at *d* = 50 nm. The modulation depth decreases as *d* increases and could still reach 8.4% when *d* = 500 nm. In general, the modulation effect of the NFRHT is stronger with smaller gap distances due to the near-field effect of the graphene plasmon polaritons.

**Figure 5:**
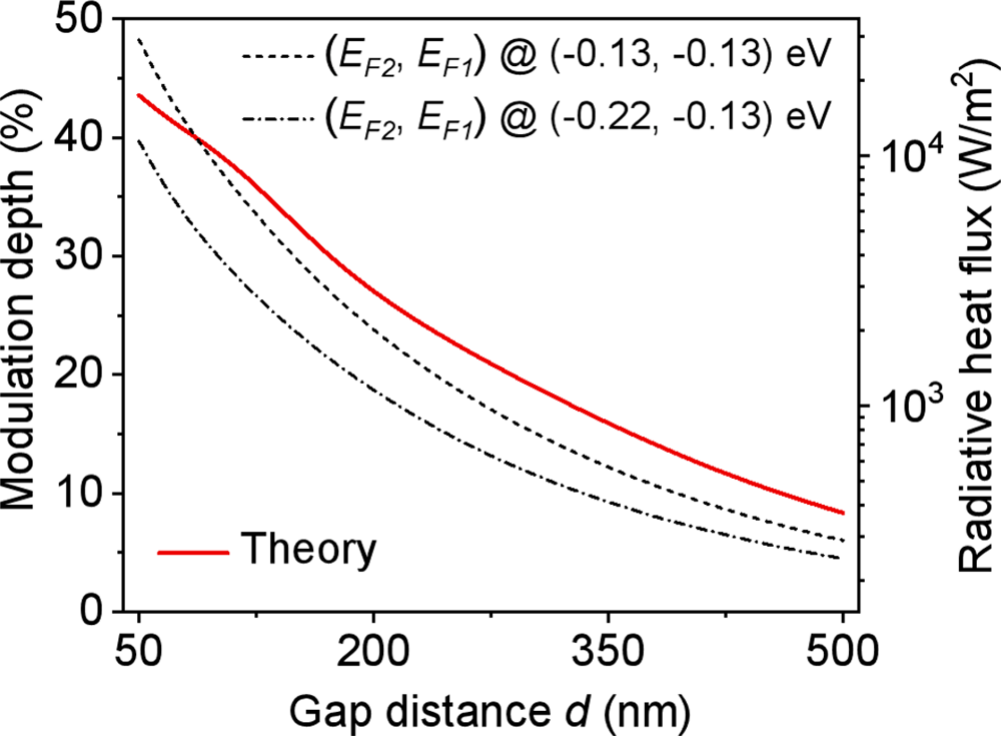
Calculated modulation depth (red-solid line) versus variable vacuum gap distance *d* within a range from 50 to 500 nm. The temperatures of the emitter and receiver are set to 308.15 and 303.15 K, respectively. The black-dashed line and dashed-dotted line show the calculated radiative heat flux between two Gr/SU8 heterostructures with graphene Fermi levels of (*E*_F2_, *E*_F1_) = (−0.13, −0.13) eV and (*E*_F2_, *E*_F1_) = (−0.22, −0.13) eV, respectively.

In conclusion, we have experimentally demonstrated an improved radiative heat flux control with an ultrahigh modulation depth of ≈32.2% with a pair of graphene-covered SU8 heterostructures at a gap distance of ≈80 nm. Tuning the graphene Fermi levels allows for coupling or decoupling of the SPP modes. Breaking the symmetry of the SPP resonance modes by changing the Fermi level of either the emitter or the receiver provides a much higher NFRHT modulation depth compared to other known methods. The theoretical analysis of the gap-dependence of the modulation depth shows that the modulation effect of the NFRHT is stronger with smaller gap distances due to the near-field effect of the graphene plasmon polaritons. This work focused on the dynamic modulation of the NFRHT by the mode mismatching method and is important for the fundamental understanding of the tunable collective optoelectronic phenomena for light–matter interaction and heat transfer at the nanoscale. The method is feasible for other van der Waals’ heterostructures with resonance modes sensitive to external stimuli. The experimental results obtained in this work provide strong evidence on mode-mismatch-induced ultrahigh dynamic NFRHT modulation with polariton-mediated materials in the near-field systems, which is of fundamental interest in polaritons control and energy manipulation. This result should inspire potential applications such as thermal switches and thermal communication.

## Supporting Information Available

Back-gated tuning of the graphene Fermi level; sample fabrication and characterization; experimental setup and measurement of the thermal resistance *R*_*x*_; gap distance between the emitter and receiver; heat conduction of the SU8 nanopillars; uncertainty analysis of the measured radiative heat flux (PDF)
