# Supplementary material for: Near-Field Radiative Heat Transfer Modulation with an Ultrahigh Dynamic Range through Mode Mismatching
Source: Nano Lett. 2022 Sep 26;22(19):7753–60. doi: 10.1021/acs.nanolett.2c01286 (PMC9562469; doi:10.1021/acs.nanolett.2c01286)
Supplement: Supplementary file 1 — nl2c01286_si_001.pdf [file nl2c01286_si_001.pdf]

Supporting Information

Near-Field Radiative Heat Transfer Modulation with an Ultrahigh Dynamic Range through Mode Mismatching

Kezhang Shi[1], Zhaoyang Chen[2], Yuxin Xing[1,3], Jianxin Yang[2], Xinan Xu[1], Julian S. Evans[1], and Sailing He[1,3,4]*

[1]Centre for Optical and Electromagnetic Research, National Engineering Research Center for Optical Instruments, Zhejiang University, Hangzhou 310058, China

[2]Centre for Optical and Electromagnetic Research, ZJU-SCNU Joint Center of Photonics, South China Academy of Advanced Optoelectronics, South China Normal University, Guangzhou 510006, China

[3]Shanghai Institute for Advanced Study, Zhejiang University, Shanghai 201203, China

[4]Department of Electromagnetic Engineering, School of Electrical Engineering, Royal Institute of Technology, Stockholm S-100 44, Sweden

E-mails: sailing@kth.se

TABLE OF CONTENTS

Section 1. **Back-gated tuning of the graphene Fermi level**

Section 2. **Sample fabrication and characterization**

Section 3. **Experimental setup and measurement of the thermal resistance R_x**

Section 4. **Gap distance between the emitter and receiver**

Section 5. **Heat conduction of the SU8 nano pillars**

Section 6. **Uncertainty analysis of the measured radiative heat flux**

Section 1. Back-gated tuning of the graphene Fermi level

According to the literature for back-gated tuning devices, the graphene Fermi level (unit: eV) of the emitter (receiver) could be obtained by:[1-3]

$$E_F = sgn(n)\hbar V_F(\pi\ |n|)^{1/2}/e, \quad \text{(S1)}$$

where sgn(x) is the sign of x, and $V_F = 1 \times 10^6$ m/s is the Fermi velocity of graphene. n (unit: cm^{-2}) is the excess-electron surface concentration of graphene, and is given by:

$$n = \eta_c(V_g - V_n), \quad \text{(S2)}$$

Here, η_c (unit: cm^{-2} V^{-1}) is a coefficient related to the capacitance-like structure (Figure 3b in the main text) and is calculated by:

$$\eta_c = C_{sum}/e, \quad \text{(S3)}$$

where C_{sum} (unit: F cm^{-2}) is the combined capacitance of C_{SU8} and $C_{\mathrm{SiO_2}}$ connected in series, calculated by:

$$C_{sum} = (C_{\mathrm{SU8}}C_{\mathrm{SiO_2}})/(C_{\mathrm{SU8}} + C_{\mathrm{SiO_2}}). \quad \text{(S4)}$$

where C_{SU8} and $C_{\mathrm{SiO_2}}$ are the capacitances per unit area of the SU8 spacer and SiO_2 dielectric, respectively. The relative dielectric constants of the SU8 and SiO_2 are 3.28 (from the producer https://kayakuam.com/wp-content/uploads/2020/07/KAM-SU-8-3000-Datasheet-7.10-final.pdf) and 3.9,[1, 3] respectively.

The graphene Fermi level E_F was calculated to be -0.205 eV without external bias voltages according to the measured radiative heat flux, which corresponds a charge-neutral-point voltage V_n of ~ 58.3 V, indicating a hole doping of the graphene sheets. When other bias voltages were applied, E_F could be obtained by Eq. (S1) and (S2). Figure S1 shows the relationship between the bias voltages V_g and the graphene Fermi levels E_F. Blue-stars represent all the cases in the main text, where V_g (i.e., V_1 or V_2) of

-10 V, 0 V, 10 V, 20 V, 35 V, 45 V correspond to E_F of -0.22 eV, -0.205 eV, -0.187 eV, -0.155 eV, -0.13 eV, and -0.098 eV, respectively. The robustness of the prediction of the graphene Fermi levels was also demonstrated by the good agreement of our computed and experimental data of the radiative heat flux in Figure 4a and 4c in the main text.

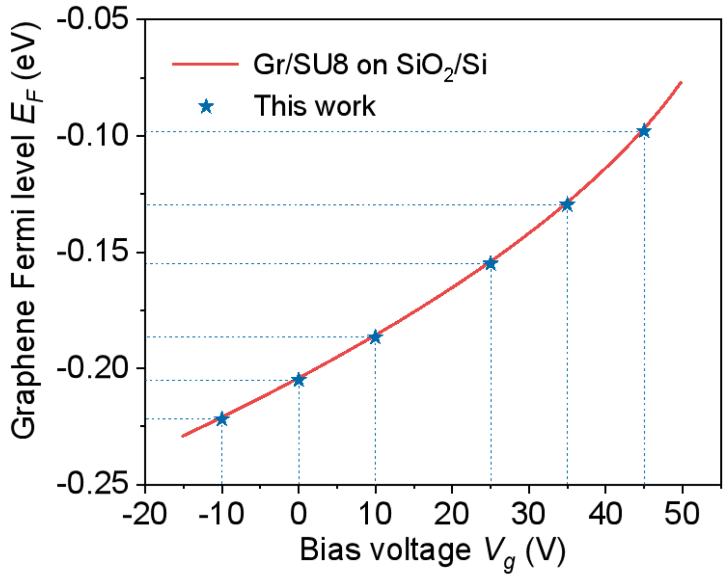

Figure S1. Calculated relationship between graphene Fermi levels and the external bias voltages of our back-gated tuning device. Blue-stars represent the cases in this work.

Section 2. Sample fabrication and characterization

Samples are fabricated using a standard UV lithography technique and wetting transfer method (for graphene sheets) as reported previously.[4] A 25 × 25 mm^2 SiO_2/Si sample was prepared with 16 × 16 mm^2 SiO_2 at the center via the UV lithography technique and inductively coupled plasma (ICP) method. A 10 × 10 mm^2 SU8 spacer with thickness of ≈ 90 nm was fabricated with dilute SU8-3005 photoresist (mass ratio, SU8 : solvent cyclopentanone = 1 : 13). Single-layer graphene with paraffin protective layer was transferred onto the SU8 spacer by wetting transfer method.[4, 5] To fabricate the Au/Ti electrodes, a 5-nm-thick titanium adhesion layer was deposited on the sample, followed by the deposition of a 95-nm-thick Au film, using an E-beam evaporation method. Eight identical SU8 nano pillars with each diameter of 20 μm were then fabricated at the center of the sample. The sample was finally cut to a required size (≈ 25 × 3 mm^2) as the receiver. The emitter sample was fabricated with the identical method without the SU8 nano pillars. The active area between the emitter and receiver was ≈ 3 × 3 mm^2. All fabrication processes were operated in a 100-class modular cleanroom.

The surface curvature of the samples measured by a laser interferometer (ZYGO OMP-035/M) could be less than ~ 10 nm within an area of 3 × 3 mm^2, which is comparable to the flatness reported elsewhere.[6-8] The samples were then considered as a flat plate-to-plate model in our calculation.

The graphene sheets used for our experiments are all single layer. Raman spectra of the graphene sheet was used to confirm the layer number of the graphene. As shown in Figure S2. the typical single-layer property of the graphene sheet was confirmed by the higher 2D mode (compared to the G mode).[4, 6, 9-11] The small D mode to G mode intensity ratio also demonstrates the high quality (i.e., little defect and good uniformity) of the graphene sample.[6, 10] The uniformity of the graphene layer was also intuitively

confirmed by both the images of the optical microscope and the atomic force microscope (AFM). The optical image of the Gr/SU8 heterostructures (Figure S3) shows the good uniformity of the graphene sheet. In addition, the heights of the surface residues of the samples characterized by AFM in Figure S4 were less than ~ 50 nm, which was smaller than the gap distance of ~ 81 nm.

The thickness of the SU8 spacers is measured by a film thickness measuring instrument (Filmetrics, F40-UV). Twenty-five random points were measured within the active area (3×3 mm^2) for the SU8 spacer[4] (not shown here). Data for each point was characterized by the reflectance from wavelength of 400 nm to 1100 nm. The standard deviation is less than 3 nm, which represents a flat surface obtained by the spin coating procedures. The minimum and maximum thickness of the SU8 films for different samples are ~ 88 nm and ~ 92 nm, respectively. In our calculation, the average value of 90 nm is employed. Figure S5 shows one of the measurements of the thickness of the SU8 spacer, indicating the agreement between the fitting and measured value.

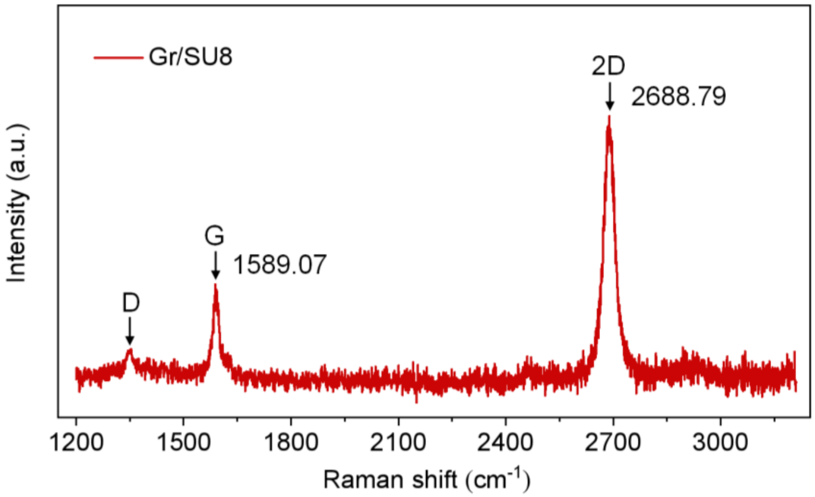

Figure S2. Raman spectra for the Gr/SU8 sample without bias voltage. The higher intensity of the 2D mode (compared to the G mode) is indicative of single-layer graphene. The small D mode to G mode intensity ratio indicates the high quality of the graphene sample.

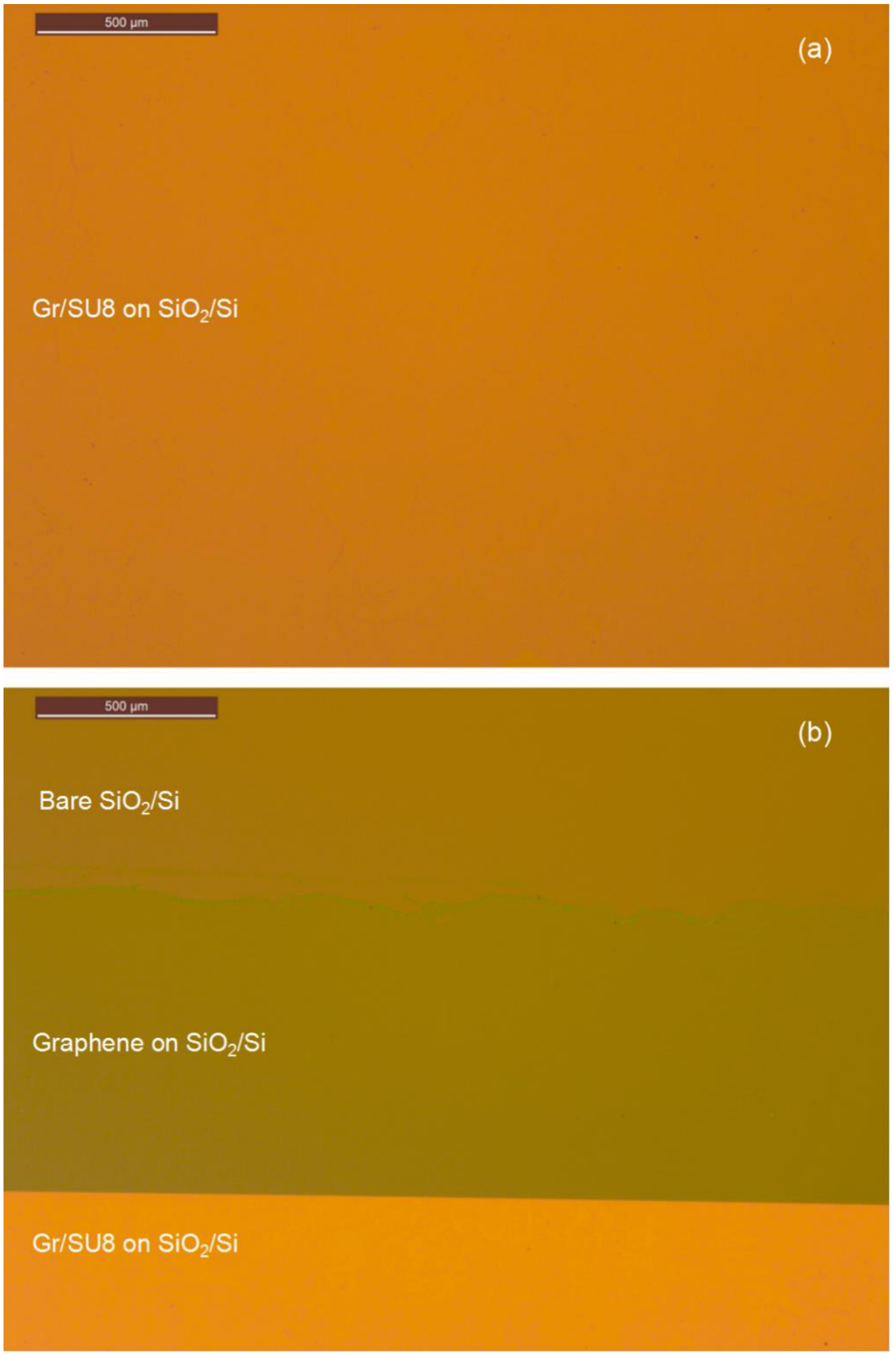

Figure S3. Optical microscope image of the Gr/SU8 samples. (a) Optical image of the graphene-covered SU8 on the SiO_2/Si substrate shows the good quality and uniformity of the graphene transfer. (b) Optical image of the sample at the edge of the SU8 spacer. The area of the graphene sheet was slightly larger than that of the SU8 spacer for better transferring. Scale bars are 500 μm in both (a) and (b).

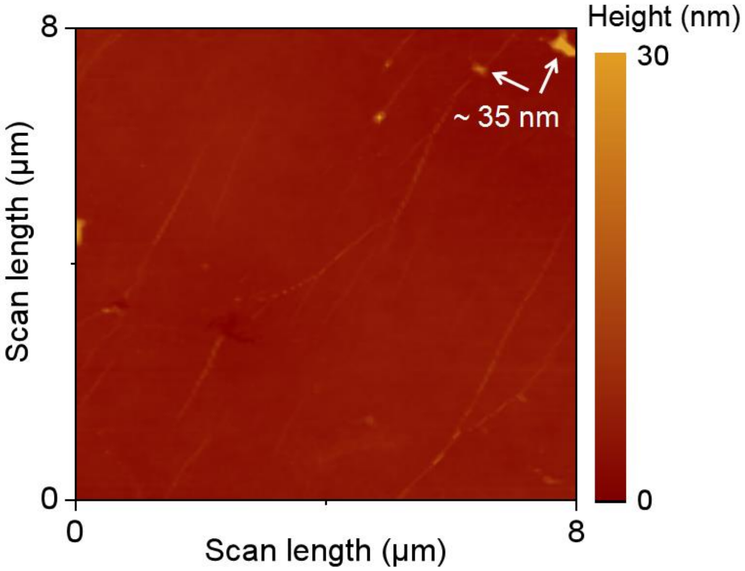

Figure S4. Typical 8 × 8 μm^2 AFM scanning image of graphene-covered SU8 heterostructure.

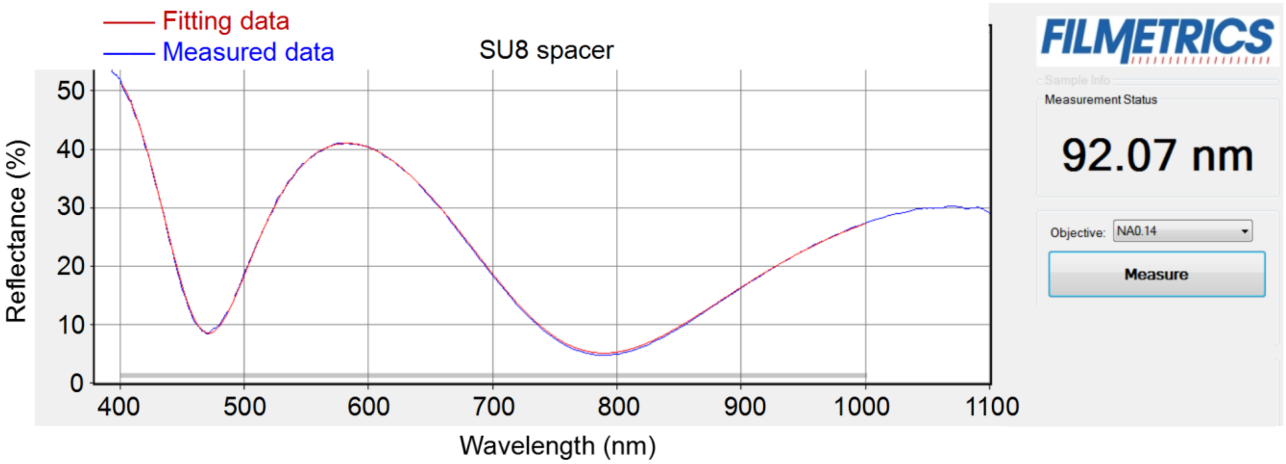

Figure S5. Thickness of each SU8 spacer obtained by the reflectance by film thickness measuring instrument (Filmetrics, F40-UV). Red line and blue line are the fitting data calculated by Cauchy model and the measured data, respectively.

Section 3. Experimental setup and measurement of the thermal resistance R_x

The home-built experimental setup consists of an emitter and receiver separated by the SU8 nano pillars. Figure 3d in the main text shows the photo of the device in a vacuum chamber at a pressure of ≈ 2.8×10^{-5} Pa. A mass of 95 g (including copper carrier of the emitter) was used to strengthen the contact and mechanical stability of the system. The heater embedded in copper carrier was used to control the temperature T_1 for the emitter. The heat flowing from the emitter to the receiver (loaded on another copper carrier) was absorbed by the heat sink through an embedded temperature electric controller (TEC), which helps to control the temperature T_2 for the receiver. Two embedded thermistors (M222 class 1/3B, Heraeus, Germany) were used to directly measure the backside temperatures (T_{1b} and T_{2b}) of the emitter and receiver. T_2 and T_1 were obtained by the equation: T_2 (T_1) = T_{2b} (T_{1b}) ± P_{sum} R_x, where measured value R_x of ≈ 9.2 K/W (see below) was the sum of the thermal resistances of the emitter (receiver), thermal conductive adhesive and the copper carrier (between the thermal conductive adhesive and the thermistors). The sum heat power was measured by a 20 × 20 × 0.4 mm^3 heat flux sensor (HFS, HS-20, Captec, France). The heat flux value was displayed by an external heat flux meter (HFM-8, Captec, France). The bias voltages of the emitter and receiver were controlled by external sources.

The thermal resistance R_x was measured by independent experiments based on the relationship between the measured temperature point T_{1b} (T_{2b}) on the backside of the emitter (receiver) and the heat flux obtained by the heat flux sensor:

$$R_x = (T_{1b} - T_{2b}) / 2P_{\mathrm{sum}} . \qquad \text{(S5)}$$

The main experimental setup and the equivalent thermal circuit are given in Figure S6. As the structure

of the receiver portion is identical to that of the emitter portion, the thermal resistances between two temperature points (T_{1b} and T_{2b}) are assumed to be $2R_x$. The emitter and receiver were two identical SiO_2/Si samples with wires connected to the electrodes (not shown). Their thermal resistances could be considered as the same as that of the Gr/SU8/SiO_2/Si samples, as the thermal resistance of the Gr/SU8 films could be ignored due to the large width-depth ratio. The emitter and receiver were physically connected through the thermal grease to reduce the contact thermal resistance. The different backside temperatures of the emitter (receiver) and the sum heat flux were measured at each thermal equilibrium state in a vacuum chamber at a pressure level of ~ 10^{-5} Pa. As a result, R_x with average value of 9.2 K/W was obtained and employed in this work.

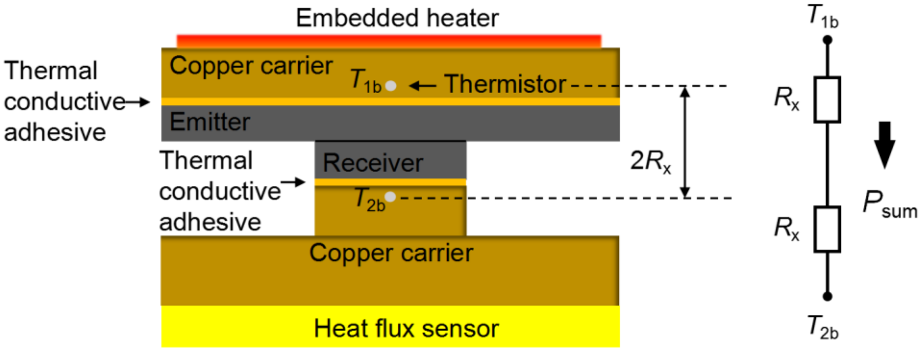

Figure S6. Schematic illustration of the main experimental setup when measuring the thermal resistances R_x. The equivalent thermal circuit is plotted on the right side. T_{1b} and T_{2b} represent temperature points on the backside of the emitter and receiver, respectively.

Section 4. Gap distance between the emitter and receiver

The gap distance between the emitter and the receiver was calculated according to the thickness of the SU8 nano pillars and the mechanical properties.[4, 8, 12] The SU8 nano pillars with diameter of 20 μm shared the identical fabrication procedures as the SU8 spacer, which allows for the uniform thickness of ~ 90 nm. In our experimental setup, a weight of 95 g was applied to the emitter for mechanical stability and a slight compression of the SU8 nano pillars was considered.[4, 8] At room temperature, the Young's modulus of SU8 is in the range of ~ 3.6 GPa to ~ 4.1 GPa.[12] For simplicity, we have taken 3.85 GPa in the calculation. A one-dimensional linear elastic analysis was employed according to Hooke's law. Displacement Δx (due to the compress) was calculated by:

$$\Delta x = h \frac{F_x}{E_{SU8}}, \tag{S6}$$

where h = 90 nm is the thickness of the SU8 nano pillar, F_x is the stress along the axial direction of the SU8 nano pillar, and E_{SU8} = 3.85 GPa is Young's modulus. According to Eq. (S6), the weight of 95 g would lead to a displacement of ~ 9 nm. Therefore, the gap distance between the emitter and receiver was ~ 81 nm (= 90 nm - 9 nm), which was slightly smaller than the original thickness of the SU8 nano pillars. The analyses have been verified with Ref. [8] computed by COMSOL Multiphysics and have been confirmed by the agreement between the computed and experimental data of the near-field radiative heat transfer (NFRHT) for a pair of SiO_2/Si samples in our previous work.[4]

Section 5. Heat conduction of the SU8 nano pillars

The heat power of the conduction from the SU8 nano pillars at temperature difference of ΔT was calculated based on Fourier's Law:

$$P_c = S\kappa \, |\Delta T| / d \, , \tag{S7}$$

where S is the sum area of eight SU8 nano pillars, d = 81 nm is the thickness of compressed SU8 nano pillars, and κ = 0.2 W m^{-1} K^{-1} is the thermal conductivity[4, 8]. In Figure S7 we have plotted the ratios of the heat conduction P_c with respect to the sum heat power P_{sum} (= $P_c + P_r$) for the Gr/SU8 samples at different E_F. The temperature difference was 5 K. The blue line shows the results when $E_{F1} = E_{F2}$ (i.e., the matched cases in the main text) while the red line shows the results of the mismatched cases when only E_{F1} = -0.13 eV. The minimum ratio was ~ 20% when $E_{F1} = E_{F2}$ = -0.13 eV and the maximum ratio was ~ 37.6% when E_{F1} = -0.13 eV and E_{F2} = -0.22 eV. Hence, the contribution of the heat conduction to the sum heat power is relatively small in our experiments. The results of the near-field radiative heat power P_r (obtained by subtracting the calculated heat conduction P_c from the measured sum heat power P_{sum}) should be close to the actual P_r.

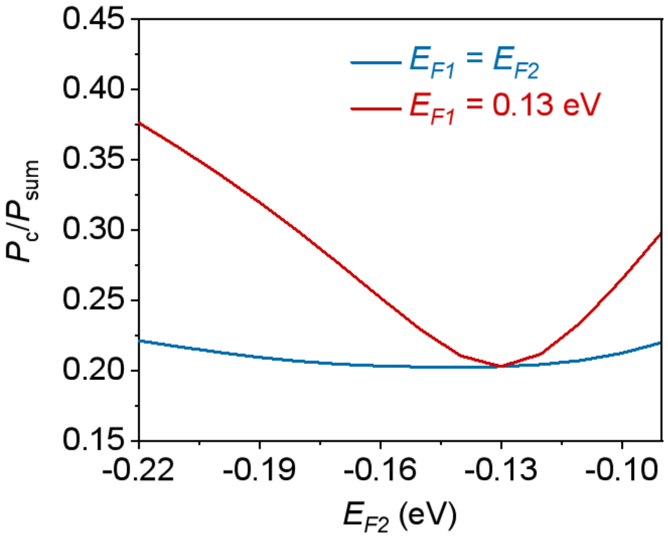

Figure S7. Ratio of the heat conduction P_c with respect to the sum heat power P_{sum} for the Gr/SU8/5L samples with different graphene Fermi levels when d = 81 nm and ΔT = 5 K. The blue line gives the results when $E_{F1} = E_{F2}$. The red line shows the mismatched cases when only E_{F1} = -0.13 eV.

Section 6. Uncertainty analysis of the measured radiative heat flux

In our case, the uncertainty of the NFRHT measurement mainly comes from the uncertainty of the active area between the emitter and the receiver (as other factors can hardly affect the results, e.g., the standard deviations of the temperature differences are quite small : ΔT = 5.04 ± 0.13 K). Since the emitter and receiver are placed in a cross shape (as shown in Figure 3c in the main text), the active area only depends on the width of the samples. Hence, the widths of the samples after cutting to a required size (length of ~ 25 mm, width of ~ 3 mm) were measured by a vernier caliper. Thirty samples were tested and the widths of the samples are at a range between 2.9 mm and 3.1 mm. Hence, the possible active area between a range from 2.9 × 2.9 mm^2 to 3.1 × 3.1 mm^2 is considered. For simplicity, three typical active areas of 2.9 × 2.9 mm^2, 3 × 3 mm^2, and 3.1 × 3.1 mm^2 were then used for the uncertainty analysis. The error bar of each point in Figure 4a in the main text is the standard deviation that comes from these three possible active areas after four measurements.

Here we give the detailed uncertainty analysis of one of the measured points. For example, when (V_2, V_1) = (35, 35) V are applied to the samples, the average value of the radiative heat power (unit: W) after four measurements is 0.1284 W. This heat power possibly comes from the active areas of 2.9 × 2.9 mm^2, 3 × 3 mm^2, or 3.1 × 3.1 mm^2. Hence, the radiative heat flux (unit: W/m^2) has possible value of 1.527 × 10^4 W/m^2, 1.427 × 10^4 W/m^2, or 1.337 × 10^4 W/m^2. The average value is 1.43 × 10^4 W/m^2 and the standard deviation (i.e., the error bar) is 0.0953 × 10^4 W/m^2. The uncertainty analyses are similar when dealing with other measured points in the main text.

References:

1. Fei, Z.; Andreev, G. O.; Bao, W.; Zhang, L. M.; A, S. M.; Wang, C.; Stewart, M. K.; Zhao, Z.; Dominguez, G.; Thiemens, M.; Fogler, M. M.; Tauber, M. J.; Castro-Neto, A. H.; Lau, C. N.; Keilmann, F.; Basov, D. N. Infrared nanoscopy of dirac plasmons at the graphene-SiO2 interface. *Nano Lett.* **2011,** 11, (11), 4701-4705.

2. Pisana, S.; Lazzeri, M.; Casiraghi, C.; Novoselov, K. S.; Geim, A. K.; Ferrari, A. C.; Mauri, F. Breakdown of the adiabatic Born-Oppenheimer approximation in graphene. *Nat. Mater.* **2007,** 6, (3), 198-201.

3. Novoselov, K. S.; Geim, A. K.; Morozov, S. V.; Jiang, D.; Katsnelson, M. I.; Grigorieva, I. V.; Dubonos, S. V.; Firsov, A. A. Two-dimensional gas of massless Dirac fermions in graphene. *Nature* **2005,** 438, (7065), 197-200.

4. Shi, K.; Chen, Z.; Xu, X.; Evans, J.; He, S. Optimized colossal near-field thermal radiation enabled by manipulating coupled plasmon polariton geometry. *Adv. Mater.* **2021,** 33, (52), 2106097.

5. Leong, W. S.; Wang, H.; Yeo, J.; Martin-Martinez, F. J.; Zubair, A.; Shen, P. C.; Mao, Y.; Palacios, T.; Buehler, M. J.; Hong, J. Y.; Kong, J. Paraffin-enabled graphene transfer. *Nat. Commun.* **2019,** 10, (1), 867.

6. Yang, J.; Du, W.; Su, Y.; Fu, Y.; Gong, S.; He, S.; Ma, Y. Observing of the super-Planckian near-field thermal radiation between graphene sheets. *Nat. Commun.* **2018,** 9, (1), 4033.

7. Ghashami, M.; Geng, H.; Kim, T.; Iacopino, N.; Cho, S. K.; Park, K. Precision Measurement of Phonon-Polaritonic Near-Field Energy Transfer between Macroscale Planar Structures Under Large Thermal Gradients. *Phys. Rev. Lett.* **2018,** 120, (17), 175901.

8. DeSutter, J.; Tang, L.; Francoeur, M. A near-field radiative heat transfer device. *Nat. Nanotechnol.* **2019,** 14, (8), 751-755.

9. Ni, Z.; Wang, Y.; Yu, T.; Shen, Z. Raman spectroscopy and imaging of graphene. *Nano Research* **2010,** 1, (4), 273-291.

10. Yan, H.; Xia, F.; Zhu, W.; Freitag, M.; Dimitrakopoulos, C.; Bol, A. A.; Tulevski, G.; Avouris, P. Infrared spectroscopy of wafer-scale graphene. *ACS Nano* **2011,** 5, (12), 9854-9860.

11. Shi, K.; Sun, Y.; Chen, Z.; He, N.; Bao, F.; Evans, J.; He, S. Colossal Enhancement of Near-Field Thermal Radiation Across Hundreds of Nanometers between Millimeter-Scale Plates through Surface Plasmon and Phonon Polaritons Coupling. *Nano Lett.* **2019,** 19, (11), 8082-8088.

12. Xu, T. G.; Yoo, J. H.; Babu, S.; Roy, S.; Lee, J. B.; Lu, H. B. Characterization of the mechanical behavior of SU-8 at microscale by viscoelastic analysis. *J. Micromech. Microeng.* **2016,** 26, (10), 105001.
